# Inverse design of anisotropic spinodoid materials with prescribed diffusivity

**DOI:** 10.1038/s41598-022-21451-6

**Published:** 2022-10-18

**Authors:** Magnus Röding, Victor Wåhlstrand Skärström, Niklas Lorén

**Affiliations:** 1 RISE Research Institutes of Sweden, Bioeconomy and Health, Agriculture and Food, Göteborg, 41276 Sweden; 2 Department of Mathematical Sciences, Chalmers University of Technology and University of Gothenburg, Göteborg, 41296 Sweden; 3 Department of Literature, History of Ideas, and Religion, University of Gothenburg, Göteborg, 40530 Sweden; 4 Department of Physics, Chalmers University of Technology, Göteborg, 41296 Sweden

**Keywords:** Computational science, Statistics, Applied mathematics, Soft materials

## Abstract

The three-dimensional microstructure of functional materials determines its effective properties, like the mass transport properties of a porous material. Hence, it is desirable to be able to tune the properties by tuning the microstructure accordingly. In this work, we study a class of spinodoid i.e. spinodal decomposition-like structures with tunable anisotropy, based on Gaussian random fields. These are realistic yet computationally efficient models for bicontinuous porous materials. We use a convolutional neural network for predicting effective diffusivity in all three directions. We demonstrate that by incorporating the predictions of the neural network in an approximate Bayesian computation framework for inverse problems, we can in a computationally efficient manner design microstructures with prescribed diffusivity in all three directions.

## Introduction

Traditionally, the search for novel materials with useful properties has relied to a large extent on experimental screening. An exhaustive search of the space of candidate material structures is then far out of reach^1^. In proportion to the rapid increases in available computational resources, a gradual move towards virtual (in silico) screening of materials and their properties has occurred, effectively circumventing this problem and accelerating materials discovery. A likely development from here is an increasing integration and cross-fertilization of not only experimental materials science and traditional numeric computation, but also statistics and machine learning, the mix sometimes being referred to as *materials informatics*^2^.

For porous materials, the microstructure, i.e. the geometry of the pore space, controls the mass transport properties^3^. Hence, quantifying such relationships is the first step towards design of materials with desirable properties. Numerous stochastic models of realistic porous microstructures found in e.g. solar cells^4^, organic semiconductors^5^, carbon electrodes^6^, platelet-filled composites^7^, lithium ion batteries^8^, mesoporous silica^9^, fiber materials^10,11^, and pharmaceutical coatings for controlled release^12^ have been developed. By computing mass transport properties like effective diffusivity and/or fluid permeability together with microstructural (geometric) descriptors, microstructure-property relationships have been established using analytical models or machine learning-based regression. Frequently used microstructural descriptors are porosity and specific surface area, tortuosity^12^, constrictivity^13^, and two-point correlation functions^14^. To name a few large-scale studies, Linden et al.^15^ predicted permeability of 536 granular materials using loglinear regression; Stenzel et al.^16^ predicted effective conductivity (mathematically analogous to effective diffusivity) of 8,119 porous network structures using conventional regression, random forests, and artificial neural networks (ANNs); Röding et al.^17^ predicted permeability of 30,000 granular as well as Gaussian random field- and spinodal decomposition-based bicontinuous structures, using conventional regression and ANNs; Prifling et al.^18^ predicted effective diffusivity and permeability of 90,000 structures with widely varying morphologies using analytical formulae, ANNs, and convolutional neural networks (CNNs). Of particular interest for this work, CNNs have been used multiple times to predict both effective diffusivity and permeability^19–22^.

Predicting effective properties of a microstructure is a ’forward’ problem with a unique solution (disregarding inaccuracy or randomness of the computation of the properties). The inverse problem, to find or design a microstructure with prescribed effective properties, i.e. inverse design, is more difficult and does in general not have a unique solution. There are in principle two classes of approaches for solving the inverse problem: (i) optimization techniques, where a solution is iteratively improved until a convergence criterion is met (sometimes combined with machine learning-based prediction of effective properties to speed up the optimization), and (ii) generative approaches, where machine learning methods are trained to directly generate a microstructure using the target properties as input. Some examples of the first kind are graph-based optimization of microstructures for photovoltaic applications^23^, optimization of the degree of anisotropy to achieve desired directional mechanical properties^24^, microstructure design of magnetoelastic alloys to optimize elastic, plastic and magnetostrictive properties^25^, optimization of anisotropic elasticity for topology optimization^26^, and optimization of microstructures for crystal plasticity^27^. Sometimes, stochastic optimization algorithms are used, such as differential evolution or genetic algorithms^28^; other times, Bayesian statistics^27^. As for the second kind, they involve e.g. generative adversarial networks, generative invariance networks, and variational autoencoders i.e. neural network architectures that can learn to generate microstructures with appearance similar to a training set of microstructures. Such approaches have been used to very quickly generate candidate structures with properties approximating the target, with respect to e.g. photovoltaic and mechanical properties^29–33^. It is worth noting, as has been pointed out numerous times, that machine learning models cannot be assumed to extrapolate well outside of the training data domain; it can only be expected to perform well inside its domain of applicability^34^ that depends both on the training data and the architecture of the model itself. This fact imposes a limitation on the applicability of all inverse design approaches utilizing machine learning.

In this work, we are concerned with inverse design of porous (two-phase), anisotropic, random microstructures with different prescribed diffusivity in all directions. Designing anisotropic mass transport properties is of interest for numerous applications, e.g. in hygiene and wound care products for transporting liquid away from the skin. This type of inverse problem rarely has a unique solution. However, by limiting the investigation to a sufficiently constrained microstructural space, with a sufficiently low-dimensional parameterization, we can at least approach a unique solution. We consider a particular class of Gaussian random fields of which the level sets i.e. thresholded, binary structures are realistic models for phase-separated structures produced by spinodal decomposition-like processes. Such structures are useful as models for e.g. microemulsions^35^, nanoporous alloys for catalysis and energy storage^36,37^, lithium-ion battery anodes^38^, and porous polymer films for controlled release^12^. Borrowing terminology from Kumar et al.^32^ and Zheng et al.^26^, we refer to these as spinodoid, i.e. spinodal decomposition-like, microstructures. This spinodoid class has a tuneable degree of anisotropy and hence the diffusivity can to some degree be tailored in all three directions. Further, their generation is much less computationally demanding than simulating actual spinodal decomposition, facilitating faster exploration of the microstructural space. We generate a large number of microstructures and train a CNN to predict effective diffusivity in all three directions. We investigate a Bayesian formulation of the inverse problem, where the solution is expressed as a posterior distribution over the parameter space of the microstructure model. We demonstrate that by incorporating the predictions of the CNN in an approximate Bayesian computation (ABC) framework, we can inversely design microstructures with prescribed, and different, diffusivity in all three directions. To our knowledge, this is the first attempt at inverse design of anisotropic microstructures with prescribed diffusivity or other types of mass transport properties, and can be considered a proof-of-concept that is applicable to other morphologies as well.

## Results

### Inverse design approach

**Figure 1:**
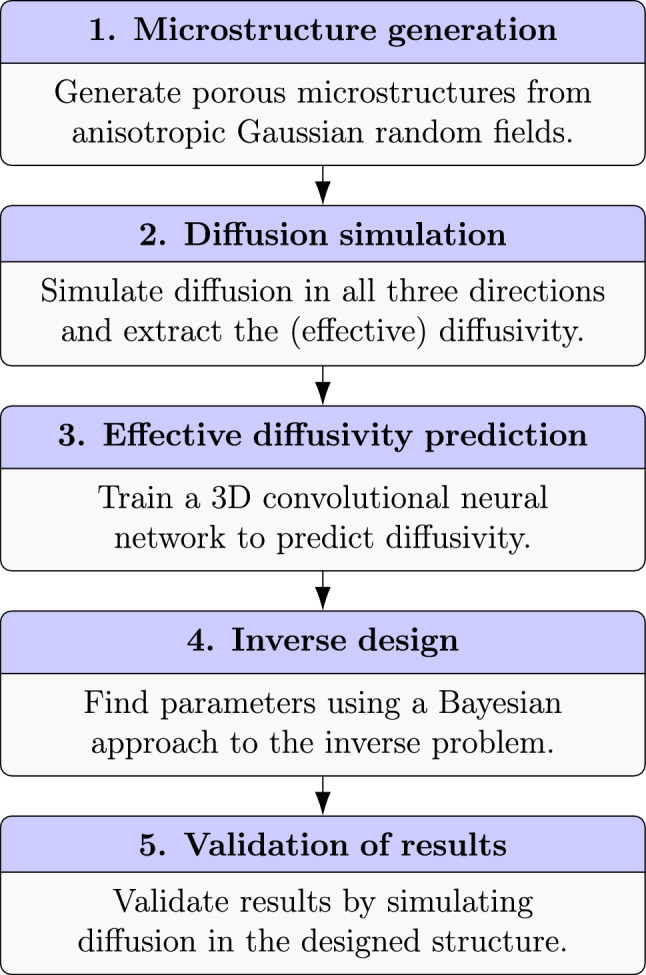
The different steps of the inverse design approach. The individual steps are explained and illustrated in the corresponding subsections.

The core concept in our inverse design approach is to utilize efficient microstructure generation and property prediction in a Bayesian framework for inverse problems to design anisotropic microstructures with prescribed diffusivity. To illustrate all the steps, we provide a schematic overview in Fig. 1. The steps of the approach are as follows: (1) we generate a large number of virtual microstructures from our model, varying parameters like porosity and degree of anisotropy; (2) the effective diffusivity is simulated in all three directions, using lattice Boltzmann methods; (3) the dataset is used to train a CNN to accurately and rapidly predict effective diffusivity (in a single direction); (4) the CNN prediction model is used as part of an approximate Bayesian computation (ABC) framework for estimating the microstructural parameters that yield the prescribed effective diffusivity; (5) the optimized structure is validated using the numerical method.

Effective diffusivity is hence computed ’offline’ i.e. before the optimization commences. It could also be computed ’online’, utilizing the numerical computation directly in the ABC framework in step 4, thereby removing the need for steps 1–3. However, this would result in the optimization being much too slow to be practically feasible, and this is precisely the reason why to introduce machine learning in the inverse design.

We elaborate on the different steps in the approach in the subsections below.

### Microstructure model

Phase separation of homogenous materials into two-phase materials by means of a diffusive process was described in the seminal paper by Cahn and Hilliard^39^. Solutions of the Cahn–Hilliard equation, expressed as the local concentration of one of the phases, $\Psi (\mathbf {x}, t)$, describes the gradual separation and coarsening of the microstructure. For a fixed time $t > 0$, the solution $\Psi (\mathbf {x})$ can be described by a superposition of cosine waves or a Gaussian random field (GRF),

1 $$\begin{aligned} \Psi (\mathbf {x}) = \sqrt{\frac{2}{M}} \sum _{m=1}^{M} \cos \left( \mathbf {q}^{(m)} \cdot \mathbf {x} + \zeta _m\right) . \end{aligned}$$

Here, *M* is large ($M \gg 1$), $\mathbf {q}^{(m)} = \left( q_x^{(m)}, q_y^{(m)}, q_z^{(m)}\right)$ are wave vectors, and $\zeta _m$ are random phase offsets in $[0, 2\pi )$. The wave vectors $\mathbf {q}^{(m)}$ are distributed according to some probability density $f\left( \mathbf {q}\right)$. If $f\left( \mathbf {q}\right)$ is radially symmetric, $\Psi (\mathbf {x})$ is statistically isotropic. A general GRF can be completely described by a mean and a covariance function^40^, and it can be shown that $f\left( \mathbf {q}\right)$ corresponds to the spectral density of the covariance function^35^. GRFs are useful models for bicontinuous two-phase microstructures, which can be obtained as a level set i.e. by thresholding: By encoding the microstructure as zeros (pore) and ones (solid) and letting

2 $$\begin{aligned} \mathcal {I}(\mathbf {x}) = \left\{ \begin{array}{ll} 1, &{} \Psi (\mathbf {x}) \ge T \\ 0, &{} \Psi (\mathbf {x}) < T \end{array} \right. , \end{aligned}$$

$\mathcal {I}(\mathbf {x})$ describes a random porous microstructure with porosity $\epsilon = P\left( \Psi (\mathbf {x}) < T\right)$. The physical counterpart to this thresholding would be the removal of one phase by e.g. leaching or dealloying.

**Figure 2:**
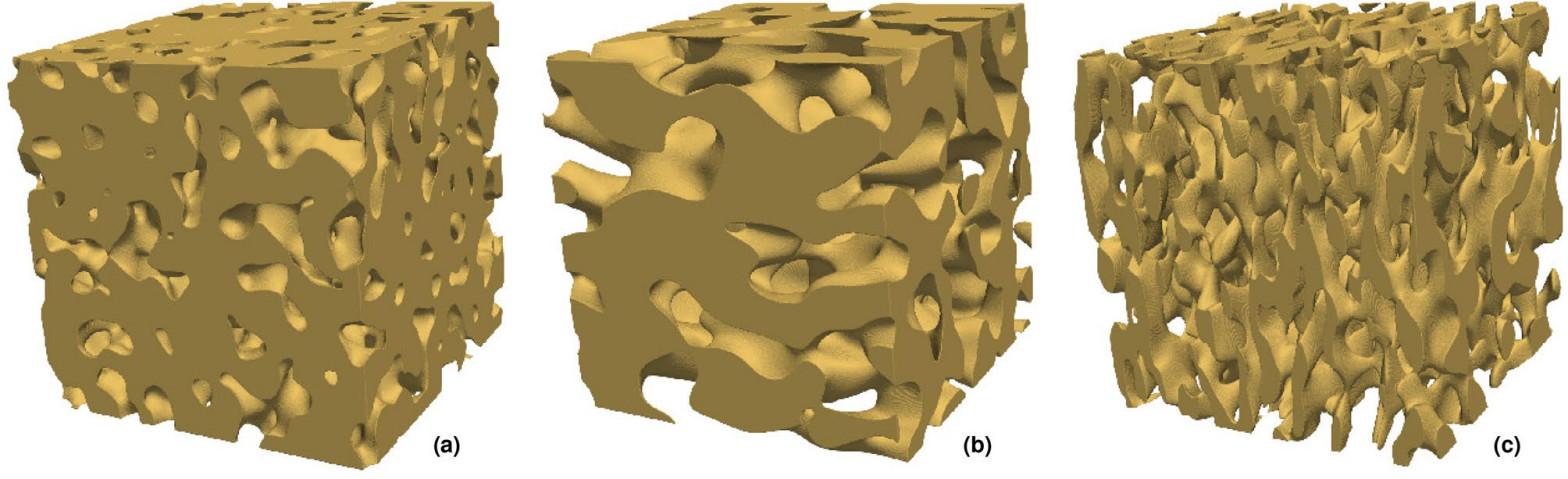
Examples of generated microstructures, with the parameters (**a**) ${\varvec{\theta }} = (0.1, 0.1, 0.1, 0.3)$ (isotropic), (**b**) ${\varvec{\theta }} = (0.15, 0.25, 0.15, 0.5)$ (longest length scale left–right), and (**c**) ${\varvec{\theta }} = (0.15, 0.05, 0.15, 0.7)$ (shortest length scale left–right).

Instead of the Cahn–Hilliard expression, we use a method based on the Fast Fourier Transform (FFT) for generation of GRFs^41^. Briefly, generating a GRF in a cubic, discrete domain $\Omega$ with resolution $N^3$ grid points ($\Omega = \{0, 1, \ldots , N-1\}^3$, say) is done in the following fashion. First, independent, normal distributed noise is generated in the spatial domain and transformed to the Fourier domain. Second, it is multiplied by the square root of the spectral density of the covariance function. Third, the result is inverse-transformed to the spatial domain, yielding a GRF with the specified covariance function. We use the spectral density

3 $$\begin{aligned} \gamma (\mathbf {q}) = \left[ 1 + \left( (a_x q_x)^2 + (a_y q_y)^2 + (a_z q_z)^2 \right) ^4 \right] ^{-2}, \end{aligned}$$

which is a modified version of a spectral density used before^41,42^. It incorporates variable degrees of anisotropy through the parameters $a_x$, $a_y$, and $a_z$ that directly control the length scale in their respective directions. Anisotropic spinodoid microstructures can be interpreted as the result of introducing anisotropic diffusion and/or anisotropic interface mobility into the phase separation dynamics^32^. As stated above, the spectral density of the covariance function can be understood as a 3D probability distribution of wave vectors; the distribution of wave vector magnitudes and hence length scales can be interpreted as heterogeneity in the phase separation dynamics. The benefit of using this FFT-based approach is that the generated GRFs conform to periodic boundary conditions of the domain $\Omega$, whereas Cahn-Hilliard type GRFs generated in a bounded domain are not periodic. For more details, see Methods. The final step to produce a porous microstructure from a GRF is thresholding to obtain a porosity $\epsilon$ as described above. Hence, the parameter vector describing the microstructure is ${\varvec{\theta }} = (a_x, a_y, a_z, \epsilon )$. However, the microstructure also depends on the random seed $\omega$ (that controls the white noise in GRF generation), and a structure is only uniquely defined by specifying $\left( {\varvec{\theta }}, \omega \right)$. In Fig. 2, examples of microstructures generated using this approach are shown, with varying porosity and degrees and directions of anisotropy.

### Dataset generation

For developing a machine learning-based prediction of diffusivity, a large number of GRF-based microstructures are generated on a grid of size $N^3$ with $N = 192$, represented as a 3D binary voxel array. The parameters are randomly sampled in suitable ranges such that the pores are sufficiently large (ensuring that reliable diffusivities are obtained from the simulations), that the inlet and outlet (i.e. the opposing edges of the structure) of the pore phase is connected in all three directions (ensuring that diffusive transport is possible), and that the same holds for the solid phase (ensuring that the microstructure is physically plausible). To this end, it is ascertained that all length scale parameters are in a suitable range, that their ratios are not ’too extreme’, and that the porosity is not too low.

Specifically, the parameters of the model are randomized in the following fashion: Let $\beta$ be the ratio of the largest and the smallest *a* value and let $\alpha$ be the ratio of the middle to the smallest *a* value. Sample $\beta$ from $\mathcal {U}[1, 3]$ (uniform distribution in [1, 3]), and sample $\alpha$ from $\mathcal {U}[1, \beta ]$. Rescale the resulting vector $\left( 1, \alpha , \beta \right)$ such that its mean value is $\mathcal {U}[0.05, 0.2]$, and assign a random permutation of these values to $a_x$, $a_y$, and $a_z$. The first step yields suitable ratios of length scales (degrees of anisotropy) and the second step yields a suitable distribution of average length scales. Then, sample the porosity $\epsilon$ from $\mathcal {U}[0.3, 0.85]$. A GRF is then generated using these parameters for a particular random seed. Before a candidate structure is accepted into the dataset, it is verified that the connectivity conditions are met. However, the parameter sampling procedure ensures a low rejection rate.

The parameters ranges further impose a limitation on how anisotropic the structures can be, as elaborated upon in the end of this section. Also, the distributions of some parameters in the final dataset are slightly skewed compared to the distribution they are originally sampled from due to rejection. In total, 8192 microstructures are generated for the training dataset, and 2048 microstructures are generated for each of the validation and test datasets. The code is implemented in Matlab (Mathworks, Natick, MA, US). Microstructure generation on an AMD Epyc 7542 CPU (2.9 GHz; single thread) takes on average 2.6 s with an additional 1.2 s for the checks.

For all structures and all three directions, diffusion simulations are performed using the lattice Boltzmann method^43,44^. The simulations are so-called forced diffusion simulations where the concentrations at the inlet and outlet are fixed with $c_\mathrm {inlet} > c_\mathrm {outlet}$, driving diffusive transport from inlet to outlet. The diffusive flux $\mathbf {J} = \left( J_x, J_y, J_z\right)$ (the net amount of transport per unit area and unit time in all directions), can be expressed

4 $$\begin{aligned} \mathbf {J} = - D \nabla c, \end{aligned}$$

where *D* is the ’free’ diffusion coefficient and $\nabla c$ is the concentration gradient. The concentration evolution is governed by the diffusion equation (Fick’s second law),

5 $$\begin{aligned} \frac{\partial c}{\partial t} = D \Delta c, \end{aligned}$$

subject to zero-flux boundary conditions at the solid-liquid interface and the fixed inlet and outlet concentrations. The effective diffusion coefficient can be computed at steady state as

6 $$\begin{aligned} D_{\mathrm {eff}, i} = - \frac{\langle J_i \rangle (N - 1)}{c_\mathrm {inlet} - c_\mathrm {outlet}}, \end{aligned}$$

where $i = x, y, z$ and $\langle J_i \rangle$ is the average flux over the simulation domain. We are interested in the ratio of $D_{\mathrm {eff}, i}$ and *D*,

7 $$\begin{aligned} \eta _i = \frac{D_{\mathrm {eff}, i}}{D}. \end{aligned}$$

This dimensionless quantity $\eta _i \in (0, 1)$ is called diffusivity (or effective diffusivity, or obstruction factor), and depends only on the geometry of the pore space, not on e.g. viscosity of the medium. The vector ${\varvec{\eta }} = (\eta _x, \eta _y, \eta _z)$ denotes the diffusivity in all three directions.

For these simulations, the solid-liquid interface has to be a triangulated surface in STL format. The computations are performed in a cluster environment using other CPUs, but for comparison we report the mean execution time for the same CPU as above: STL file generation takes on average 40 s (single thread). Simulating diffusion in one direction (64 threads) takes on average 240 s. Converting this latter result to three directions, it takes on average 720 s, and if executed on a single thread (neglecting parallelization overhead, just multiplying the time by 64), it would take on average 12.7 h. The STL generation and diffusion simulation are performed using proprietary software developed in C++^43,44^. The execution time for the simulations is almost independent of the parameters, whereas the execution time for STL generation is approximately linearly proportional to the specific surface area which in turn is inversely proportional to the length scale(s).

**Figure 3:**
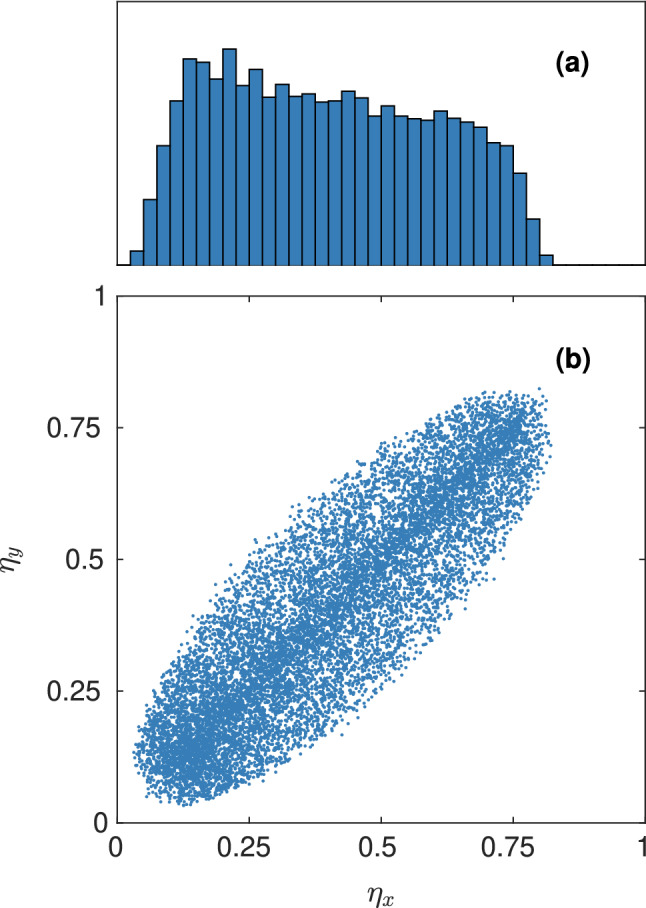
The distribution of diffusivity, showing (**a**) a histogram of the distribution of $\eta _x$ and (**b**) a scatter plot of the joint distribution of $\eta _x$ and $\eta _y$. Because the length scale parameters in all directions are distributed equally, the distributions look the same for any two directions. Note that the x axis is the same for (**a**) and (**b**).

It is of particular interest what the joint distribution of diffusivity in the three directions is i.e. what combinations of (one-dimensional) diffusivity are represented in the dataset. The size and resolution of the simulation domain limits the length scale both downward and upward. Also, assuming a fully-connected pore space, the porosity and specific surface area will be the same for transport in all three directions, and because they have substantial influence on transport, the diffusivity in different directions will be correlated. Of course, the differences increase with increasing anisotropy, but the degree of anisotropy can in practice not be too extreme because then the requirement of a connected pore space would be violated with high probability. In Fig. 3, the distribution of diffusivity is illustrated. Indeed, the diffusivity in different directions is strongly positively correlated ($r \approx 0.88$).

### Prediction of diffusivity

We implement a convolutional neural network (CNN) for prediction of diffusivity in a single direction. In a conventional artificial neural network (ANN), the building blocks are fully-connected layers that each comprise a number of nodes. Weighted sums of the outputs from the nodes in one layer form the inputs to the next layer, and a so-called activation function *f* is applied to introduce non-linearity. In contrast, in a CNN, convolutional layers are the main building blocks. A typical CNN architecture consists of both convolutional layers, pooling layers, and fully-connected layers. In a convolutional layer, the input is convolved with several convolution kernels and an activation function *f* is applied to produce outputs known as feature maps. In a pooling layer, feature maps are downsampled by computing e.g. the mean or maximum on small patches of the feature maps from the preceding layer. After the convolutional and pooling layers, fully-connected layers are used to compute the final (scalar) output. During training of a CNN, the parameters (convolution kernel elements and weights of the sums in the fully-connected layers) are optimized with respect to minimizing deviations from the target output measured by some loss function^45,46^.

It is worth mentioning that the alternative approach to using a CNN would be to extract microstructural descriptors such as porosity, specific surface area, tortuosity, and correlation functions, and use them as input for basically any machine learning method e.g. a conventional ANN. However, considering the computational demand of extracting such descriptors, this approach would in practice be much slower than using a CNN (even with a very small ANN). We investigated ANNs in this project using such descriptors, obtaining almost the same predictive performance as for the CNN but with much longer execution times (not shown).

**Figure 4:**
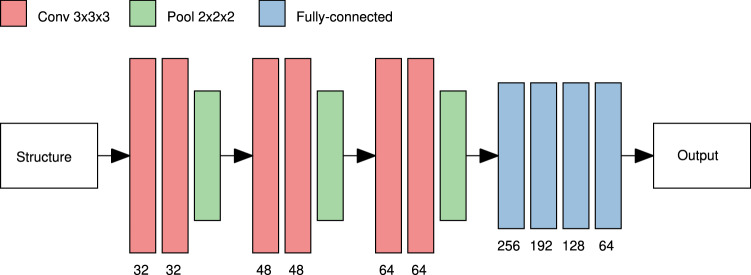
Illustration of the CNN architecture. Arrays of size $96^3$ are used as input. First, three convolutional blocks having convolutional layers with 32, 48, and 64 filters and average pooling layers are used to extract microstructural features at different scales. Then, four fully-connected layers with 256, 192, 128, and 64 nodes are used to produce the logit-transformed diffusivity prediction as output. Elu activation is applied after all convolutional and fully-connected layers.

We design a CNN that produces a diffusivity prediction as output given a microstructure as input. To reduce the computational workload and accelerate training, the microstructures are downsampled by a factor of 2 and stored as $96^3$ arrays with values in $\{0, \ldots , 8\}$ (the values representing the number of solid voxels in all non-overlapping windows of size $2 \times 2 \times 2$ in the original structures). Preprocessing the microstructures in this manner is equivalent to applying an average pooling filter with a $2 \times 2 \times 2$ window as the first layer in the CNN, apart from a linear scaling. The CNN comprises three convolutional blocks, each with two convolutional layers and one average pooling layer. For the convolutional layers, $3 \times 3 \times 3$ kernels and 32, 48, and 64 filters per layer are used in the different blocks. For the average pooling layers, $2 \times 2 \times 2$ windows are used. After the convolutional part, 4 fully-connected layers with 256, 192, 128, and 64 nodes are used to compute the final output. The network has approximately 8.8M weights. After all convolutional and fully-connected layers, the exponential linear unit (Elu) activation^47^,

8 $$\begin{aligned} f(x) = \left\{ \begin{array}{ll} x, &{} x > 0 \\ \alpha \left( e^x - 1\right) , &{} x \le 0 \end{array} \right. , \end{aligned}$$

is used for $\alpha = 1$ (the only value for which the Elu activation is once-differentiable). The convolutional blocks can be thought of as feature extractors that extract microstructural descriptors at different scales, which are then used as input to the fully-connected part. The inputs are rescaled to $[-1/2, 1/2]$, because normalization of inputs tend to provide faster convergence in training^48^. The outputs are logit-transformed so that the network in fact is trained to predict $y = \log (\eta /(1-\eta ))$. The network architecture is illustrated in Figure 4.

**Figure 5:**
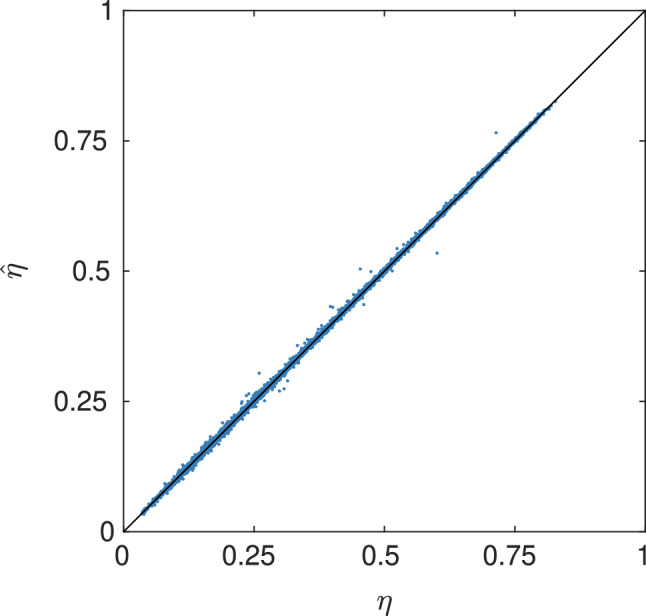
True diffusivity $\eta$ vs predicted diffusivity $\hat{\eta }$ for the test set, including all three directions.

The networks are implemented in Tensorflow^49^ and optimized with respect to mean squared error (MSE) loss in the logit scale. The CNN is trained to predict diffusivity in only one direction. To account for all three directions, each microstructure is included three times in the datasets, with permuted axes. Therefore, the dataset sizes used for CNN training and testing are three times larger than the corresponding number of microstructures i.e. 24,576 for the training dataset and and 6144 each for the validation and test datasets. The reason for predicting a single diffusivity instead of all three at once is that it enables more data augmentation. Training is run for 3500 epochs on a single NVIDIA A100 GPU, and the execution time is approximately 14 days (340 s per epoch). It is worth noting that whereas training of CNNs used for classification tasks would typically converge in approximately 10–100 iterations, training of CNNs used for regression tasks frequently require a much larger number of epochs to converge. The model yielding the minimal validation loss over all epochs is selected. For more details on the training procedure and the data augmentation scheme, see Methods. In Table 1, the best results are shown. In Fig. 5, the true and predicted values are compared for the test set.

**Table 1:** Error measures for the prediction of diffusivity, where MSE and MAPE (in %) is given for the training, validation and test sets. Note that MSE is evaluated on the logit scale and MAPE on the linear scale.

	Training	Validation	Test
MSE	$3.5766 \cdot 10^{-4}$	$4.7425\cdot 10^{-4}$	$5.0987\cdot 10^{-4}$
MAPE	0.8689	0.9615	0.9986

### Optimization and inverse design

The final step is to design microstructures with prescribed diffusivity, and efficiently explore the space of candidate microstructures. As mentioned in the Introduction, the prediction model will only perform well inside its domain of applicability^34^. The small test data losses indicate that the domain of applicability at least approximately covers the space of microstructures defined by the dataset generation procedure. Therefore, using the CNN prediction model as part of an inverse design scheme should be ’safe’ within this space. However, even within the domain of applicability, the error relative to the ’ground truth’ i.e. the lattice Boltzmann method can be expected to be around 1 % and possibly more, emphasizing the importance of validating the results.

#### Bayesian formulation

We use a Bayesian formulation of the inverse problem. Whereas the diffusivity vector ${\varvec{\eta }} = (\eta _x, \eta _y, \eta _z)$ is entirely determined by the microstructure, i.e. $\mathcal {I}(\mathbf {x})$, we reiterate that the generated microstructures are functions of not only the parameter vector ${\varvec{\theta }} = (a_x, a_y, a_z, \epsilon )$ but also of the random seed $\omega$. Therefore, the relationship between ${\varvec{\theta }}$ and ${\varvec{\eta }}$ is stochastic. In Bayesian terms, we can express this as

9 $$\begin{aligned} f\left( {\varvec{\theta }}|{\varvec{\eta }}\right) \propto P\left( {\varvec{\eta }}|{\varvec{\theta }}\right) \pi \left( {\varvec{\theta }}\right) , \end{aligned}$$

where $P\left( {\varvec{\eta }}|{\varvec{\theta }}\right)$ is the likelihood, i.e. the probability of observing ${\varvec{\eta }}$ as the output given that ${\varvec{\theta }}$ is the input, and $\pi \left( {\varvec{\theta }}\right)$ is the prior distribution, reflecting prior knowledge of ${\varvec{\theta }}$. The posterior distribution $f\left( {\varvec{\theta }}|{\varvec{\eta }}\right)$ is the full solution to the inverse problem of finding parameters ${\varvec{\theta }}$ that yield the property ${\varvec{\eta }}$. The posterior distribution can be summarized e.g. by computing the posterior mean (the average of ${\varvec{\theta }}$) which we denote $\tilde{{\varvec{\theta }}}$.

#### Approximate Bayesian computation

The relationship between ${\varvec{\theta }}$ and ${\varvec{\eta }}$ can only be observed indirectly by simulation and prediction; in other words, the likelihood is not analytically tractable. Approximate Bayesian computation (ABC) is designed for obtaining samples from an approximate posterior distribution in this setting. The starting point of ABC is the fact that samples can be drawn from the (exact) posterior in the following fashion: if a random ${\varvec{\theta }}$ is sampled from the prior, a microstructure is generated for that ${\varvec{\theta }}$ with the predicted diffusivity ${\varvec{\eta }}'$ (we drop the ’hat’ notation for predicted values in this context), and if ${\varvec{\eta }}' = {\varvec{\eta }}$, then ${\varvec{\theta }}$ is accepted and otherwise rejected as a sample from the posterior. Because $P\left( {\varvec{\eta }}' = {\varvec{\eta }}|{\varvec{\theta }}\right)$ is virtually zero, we can accept ${\varvec{\theta }}$ as a sample from an approximate posterior if $\rho \left( {\varvec{\eta }}, {\varvec{\eta }}'\right) \le \tau$ for some discrepancy $\rho$ and tolerance $\tau$ to obtain a higher, manageable acceptance rate^50–52^. Several more computationally efficient methods than straightforward rejection sampling have been proposed such as Markov chain Monte Carlo^53^, partial rejection control^54^, sequential Monte Carlo^55^, and finally population Monte Carlo^56^ which we use herein.

Population Monte Carlo ABC is based on an analogous method for standard Bayesian inference^57^. By using a decreasing sequence of tolerances, $\tau _1$, $\tau _2$, ..., $\tau _T$, a population of *P* individuals, ${\varvec{\theta }}_1, \ldots , {\varvec{\theta }}_P$, is adapted to approximate a sample from the true posterior increasingly well. The method is adapted to our case from Beaumont et al.^56^ (see Methods).

#### Case study

We consider inverse design for two cases with target diffusivities ${\varvec{\eta }}_1 = \left( 0.40, 0.50, 0.60\right)$ and ${\varvec{\eta }}_2 = \left( 0.25, 0.375, 0.50\right)$. We use a flat prior over the parameter space defined in Dataset generation, i.e. sampling from the prior is equivalent to the sampling performed to generate the dataset for training the CNN. The discrepancy $\rho$ is

10 $$\begin{aligned} \rho \left( {\varvec{\eta }}, {\varvec{\eta }}'\right) = 100 \cdot \frac{1}{3} \left( \left| \frac{\eta _x' - \eta _x}{\eta _x}\right| + \left| \frac{\eta _y' - \eta _y}{\eta _y}\right| + \left| \frac{\eta _z' - \eta _z}{\eta _z}\right| \right) , \end{aligned}$$

i.e. analogous to the MAPE loss used for CNN performance assessment (see Methods). The population size used is $P = 512$. Further, we use a sequence of 9 log-equidistant tolerances, $\log _{10} \tau _1 = 2$, $\log _{10} \tau _2 = 1.75$, ..., $\log _{10} \tau _9 = 0$; hence, after the last iteration, the mean absolute error over all directions can be up to 1 %. Note that not only the approximate sampling of the ABC method but also the prediction error of the CNN is incorporated into the approximate posterior. The algorithm is implemented in Matlab (Mathworks, Natick, MA, US) with the trained CNN imported from Tensorflow and running a parallel implementation utilizing 128 threads. For the first case, the execution time is 6.2 h with the microstructure generation and CNN prediction code run $\sim$ 130,000 times. For the second case, the execution time is 69 h with the microstructure generation and CNN prediction code run $\sim$ 1,500,000 times. The number of trials increases approximately exponentially as a function of iteration number in both cases, due to the decreasing tolerance sequence.

**Figure 6:**
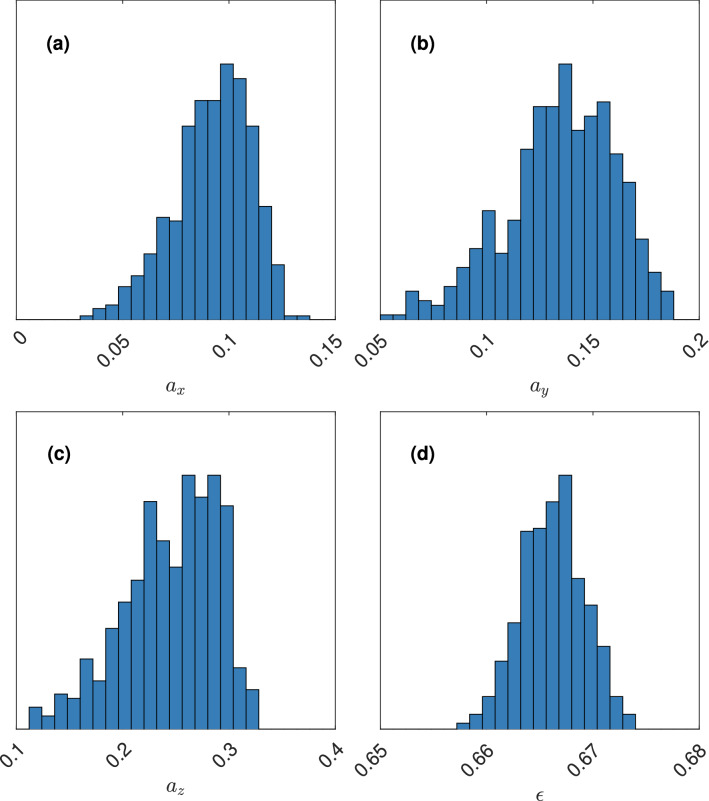
The (approximate) posterior distribution of ${\varvec{\theta }}$ for the target diffusivity ${\varvec{\eta }}_1$, showing the marginal posterior distributions of (**a**) $a_x$, (**b**) $a_y$, (**c**) $a_z$, and (**d**) $\epsilon$.

**Figure 7:**
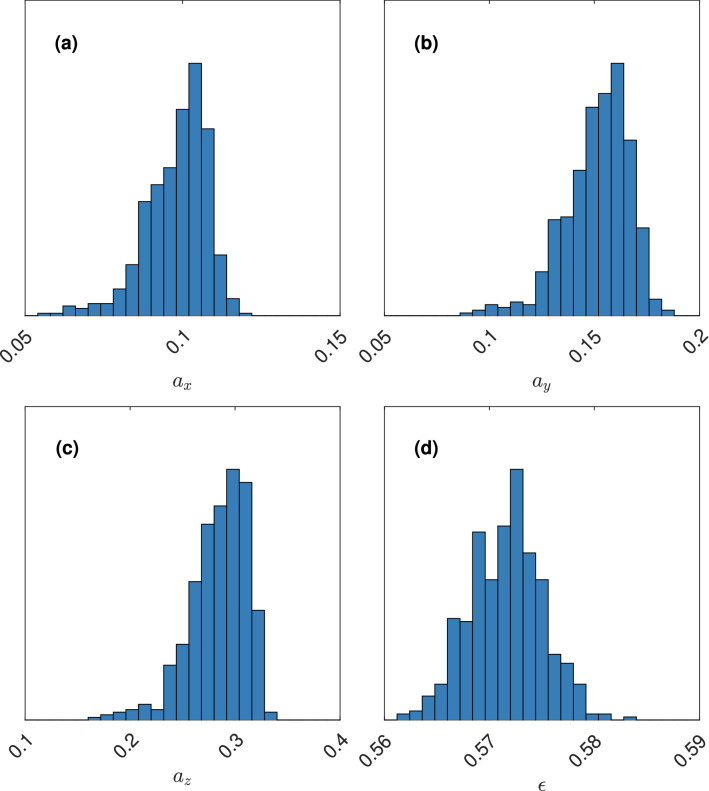
The (approximate) posterior distribution of ${\varvec{\theta }}$ for the target diffusivity ${\varvec{\eta }}_2$, showing the marginal posterior distributions of (**a**) $a_x$, (**b**) $a_y$, (**c**) $a_z$, and (**d**) $\epsilon$.

The (approximate) posterior distributions for the two cases are shown in Figs. 6 and 7.

**Figure 8:**
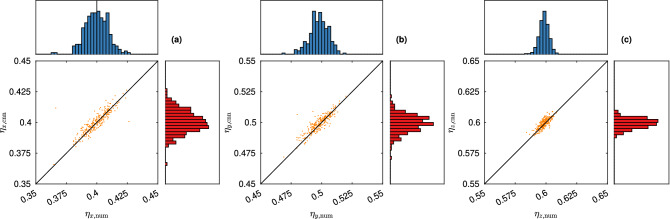
Distribution of diffusivity obtained from microstructures simulated with parameter $\tilde{{\varvec{\theta }}}_1$, showing values from the numerical method (horizontal axis/diagram) and the CNN prediction (vertical axis/diagram), for (**a**) $\eta _x$, (**b**) $\eta _y$, and (**c**) $\eta _z$.

**Figure 9:**
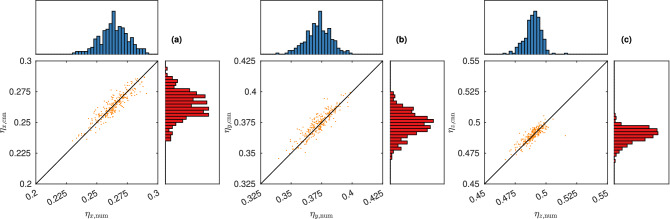
Distribution of diffusivity obtained from microstructures simulated with parameter $\tilde{{\varvec{\theta }}}_2$, showing values from the numerical method (horizontal axis/diagram) and the CNN prediction (vertical axis/diagram), for (**a**) $\eta _x$, (**b**) $\eta _y$, and (**c**) $\eta _z$.

The posterior means are $\tilde{{\varvec{\theta }}}_1 = \left( 0.092, 0.135, 0.245, 0.666\right)$ and $\tilde{{\varvec{\theta }}}_2 = \left( 0.098, 0.151, 0.284, 0.572\right)$. For these values, we generate 250 microstructures and acquire the diffusivity using both the numerical method and the CNN prediction. The results are shown in Figs. 8 and 9.

For the first case, the mean obtained diffusivity is $\left( 0.401, 0.499, 0.599\right)$ (CNN prediction) and $\left( 0.400, 0.498, 0.598\right)$ (numerical method). For the second case, the mean obtained diffusivity is $\left( 0.264, 0.374, 0.490\right)$ (CNN prediction) and $\left( 0.264, 0.372, 0.489\right)$ (numerical method). It is clear that the results are somewhat better for case 1 than for case 2. This is not a surprise, considering that posterior sampling is more than 10 times more computationally demanding for the latter case, which indicates that ${\varvec{\eta }}_1$ is more representative of the set of diffusivities spanned by the microstructural parameter ranges, whereas ${\varvec{\eta }}_2$ is more of an edge case.

#### Upscaling

**Figure 10:**
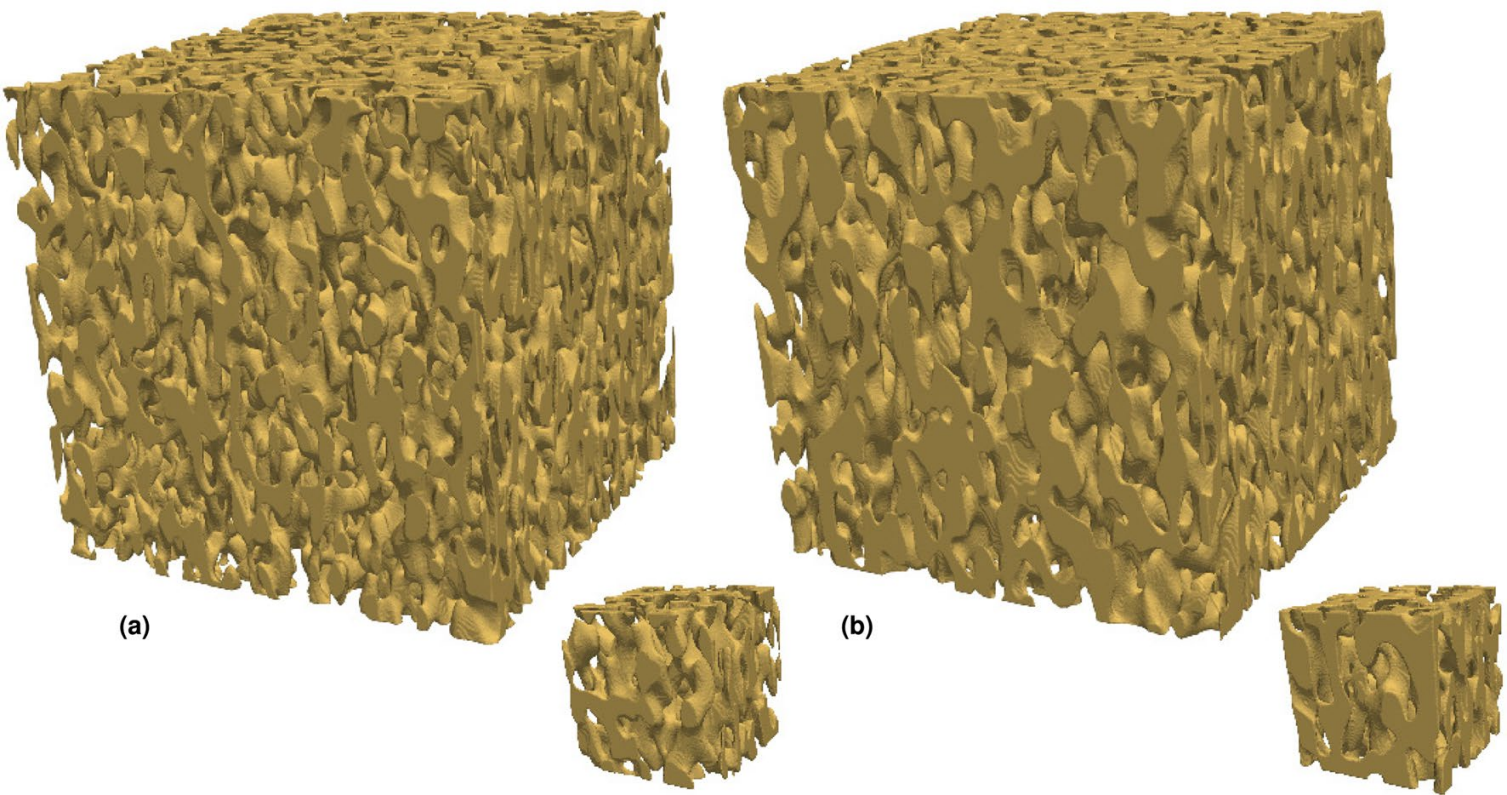
Representative structures generated using the optimized parameters, for the target diffusivities (**a**) ${\varvec{\eta }}_1$ and (**b**) ${\varvec{\eta }}_2$. Small structures ($N = 192$) in the scale the inverse design is performed is shown together with larger, upscaled structures ($N = 576$).

Whereas the inverse design procedure is executed on a rather small spatial scale, the intended outcome of inverse design is likely to identify structures with desired properties on a larger scale. We demonstrate upscaling with structures generated on a grid of size $N^3$ with $N = 576$, i.e. a factor 3 larger in all directions. Starting from the posterior mean parameter estimates $\tilde{{\varvec{\theta }}}_1$ and $\tilde{{\varvec{\theta }}}_2$ and assuming the same (albeit arbitrary) physical grid resolution as for the smaller structures, the length scale parameters $a_x$, $a_y$, and $a_z$ are divided by 3. For each case, 10 microstructures are generated, and the diffusivity is computed using the numerical method (note that in this case, the CNN prediction cannot be used because it is designed for a particular resolution). The obtained mean diffusivities are $\left( 0.393, 0.494, 0.595\right)$ and $\left( 0.258, 0.366, 0.484\right)$. Representative structures are shown in Fig. 10. Not surprisingly, a similar bias in the diffusivities is seen here as for the smaller structures. However, larger structures will have less variations in terms of diffusivity (all diffusivities have standard deviations below 0.003).

## Discussion

We present a concept for inverse design of spinodoid i.e. spinodal-like morphologies with tailored diffusivity in three directions. The microstructures are simulated as level sets of Gaussian random fields with variable anisotropy. Because the generated microstructures are random, the inverse problem of finding parameters that produces a microstructure with prescribed properties does not have a unique solution. Therefore, we use a Bayesian framework to express the solution to the inverse problem as a posterior distribution over the parameters. As part of this framework, we incorporate a CNN trained to predict diffusivity. CNN prediction is much less computationally demanding than numerical simulation, allowing for optimization of microstructures with respect to its diffusivity within reasonable time. Although it is abundantly clear from the obtained posterior distributions that there is no unique solution, we demonstrate on two cases that using the posterior mean of the microstructural parameters, this framework can be used to obtain parameters that yield structures with properties close to the prescribed values. It would be possible to validate the approach more carefully using experimental data e.g. by 3D printing of an optimized structure and performing pulsed-field gradient nuclear magnetic resonance measurements of anisotropic diffusion in the structure. However, this is beyond the scope of this work.

Although the framework is designed for relatively low resolution microstructures, we demonstrate that upscaling works by generating larger structures that are shown to have diffusivities close to the prescribed values and with smaller variance, indicating that the problem of non-uniqueness is reduced by increasing structure size.

It is worth noting that there are at least two potential sources of bias in the results. First, virtually all statistical estimation procedures introduce a bias, and the Bayesian posterior mean estimation is no exception. Second, the approximate nature of the ABC procedure is known to introduce additional bias. In summary, the proposed method to find a solution to the inverse problem produces a biased choice among the possible solutions.

The inverse design process could be further accelerated in several ways. For example, the convolutional neural network architecture could be modified to predict diffusivity in all directions jointly. Further, by training a generative network, such as a variational autoencoder or a generative adversarial network, to directly generate a candidate structure with prescribed properties, the iterative sampling and optimization procedure could be circumvented. Also, the fact that the variation in diffusivity is much smaller for larger microstructures indicate that more reliable solutions to the inverse problem can be found if the study is performed in higher resolution; this would however require very substantial computational resources, and 3D CNNs will likely not be feasible for much higher resolutions in the near future. One possible workaround is to use less computationally demanding 2D CNNs, taking e.g. average porosity maps along one axis as input.

In principle, the inverse design problem could be solved by using e.g. a conventional ANN to establish a mapping from the diffusivity vector to the parameter vector. The CNN as well as the ABC framework would then not be needed. However, the relationship between the parameter vector and the resulting microstructure is inherently random, introducing uncertainty in the solution of the inverse problem. Therefore, machine learning is more straightforward to use for approximating the deterministic ’forward’ problem of predicting diffusivity from the microstructure, combined with a statistical framework for quantifying uncertainty. This is precisely the motivation of our approach.

To our knowledge, this is the first attempt at inverse design of anisotropic microstructures with prescribed diffusivity or other types of mass transport properties. The approach can be considered a proof-of-concept that is applicable to other morphologies as well e.g. anisotropic fibers or anisotropic granular microstructures. Although beyond the scope of this work, the method is also applicable to other types of properties, such as more complex diffusion simulations involving finite-sized particles and adsorption. However, such simulations may not necessarily be practically feasible due to the heavy computational demands. An interesting further challenge, indeed a considerable one, is to relate the parameters of anisotropic microstructures to raw material and processing parameters for manufacturing. This is an inverse problem by itself. Finally, to facilitate further development in this area, the data and the codes for microstructure generation, training of the convolutional neural network, and inverse design are available open access.

## Methods

### Gaussian random fields

The starting point of the method for generating GRFs is that a covariance function for a GRF, $C(\mathbf {x}, \mathbf {y})$, can be expressed in terms of its spectral density $\gamma (\mathbf {q})$,

11 $$\begin{aligned} C(\mathbf {x}, \mathbf {y}) = \int e^{-2 \pi i \mathbf {q} \cdot (\mathbf {x} - \mathbf {y})} \gamma (\mathbf {q}) d\mathbf {q}, \end{aligned}$$

known as the Wiener–Khinchin formula^40^. Starting with a Gaussian white noise process *W* i.e. $W(\mathbf {x})$ is $\mathcal {N}(0, 1)$-distributed and independent for all $\mathbf {x}$ (*W* is a GRF with covariance $\delta (\mathbf {x} - \mathbf {y})$), the GRF

12 $$\begin{aligned} \Psi (\mathbf {x}) = \left( \mathcal {F}^{-1} \gamma ^{1/2} \mathcal {F}W\right) (\mathbf {x}) \end{aligned}$$

has covariance $C(\mathbf {x}, \mathbf {y})$. Note that because both $\gamma$ and the Fourier transform of *W* are symmetric along all three axes, the imaginary part of the resulting GRF is guaranteed to be zero. A detailed account is provided in Lang and Pothoff^41^, and it should be pointed out that because the FFT enforces a discrete representation of the spectral density, it is actually an approximate method, which is a necessary sacrifice to obtain a periodic structure.

### Convolutional neural network

The CNN is trained using stochastic gradient descent (SGD) with momentum 0.9 for optimization^58,59^ and a batch size of 16, the maximum size possible considering the network architecture and the available GPU memory. The learning rate *LR* is varied such that $\log _{10} LR$ is $\{-4, -3.75, -3.5, -3.25, -3, -2.75\}$ for 25 epochs each, $-2.5$ for 1,600 epochs, $-2.75$ for 875 epochs, and finally $-3$ for 875 epochs, in total comprising 3,500 epochs.

We also utilize a data augmentation scheme. Note that the diffusivity is invariant to mirroring and rotation of the microstructures orthogonal to the direction of transport. Also, given the periodicity of the microstructures, the diffusivity is invariant to circular shifts. The reason for training the CNN to predict only a single diffusivity at once is to leverage this invariance. In the training dataset, we introduce random flipping and swapping of the axes as well as circular shifts. The data augmentation increases the (theoretical) size of the training dataset by a factor of $2 \times 2 \times 2 \times 96^2 = 73,728$, acting as a powerful regularizer that improves the generalization of the CNN^60^.

Because of the considerable dataset sizes, the data are memory-mapped instead of being loaded into CPU memory. The augmentation as well as the rescaling of the inputs and transformation of the outputs are performed batchwise after loading a batch into memory.

The weights are optimized with respect to mean squared error (MSE) loss,

13 $$\begin{aligned} \mathrm {MSE} = \left\langle \left( \hat{y} - y \right) ^2 \right\rangle , \end{aligned}$$

where *y* is the target value (numerical simulated, logit-transformed diffusivity) and $\hat{y}$ is the predicted value. Using that $y = \log (\eta /(1-\eta ))$ where $\eta$ is the diffusivity, we can also write

14 $$\begin{aligned} \mathrm {MSE} = \left\langle \left( \log \frac{\hat{\eta }}{1-\hat{\eta }} - \log \frac{\eta }{1-\eta } \right) ^2 \right\rangle = \left\langle \left( \log \frac{\hat{\eta }(1-\eta )}{\eta (1-\hat{\eta })} \right) ^2 \right\rangle . \end{aligned}$$

The MSE is evaluated in the logit scale to attain a weighting of the samples that is more even and independent of $\eta$. A predicted value $\hat{y}$ is converted to a diffusivity prediction $\hat{\eta }$ by the inverse logit transform, $\hat{\eta }= 1/(1+\exp {(\hat{y})})$. For final assessment of predictive performance, we use the more intuitive mean absolute percentage error (MAPE) loss,

15 $$\begin{aligned} \mathrm {MAPE} = 100 \cdot \left\langle \left| \frac{\hat{\eta }- \eta }{\eta }\right| \right\rangle \%. \end{aligned}$$

The MSEs and MAPEs should be understood as averages over all microstructures in the dataset and over all three directions.

### Approximate Bayesian computation

The ABC method from Beaumont et al^56^ is adapted to our case. A population of *P* individuals, ${\varvec{\theta }}_1, \ldots , {\varvec{\theta }}_P$, are first initialized by sampling from the prior without constraints on $\rho$. Then, the population is iteratively updated to approximate a sample from the posterior distribution by using a decreasing sequence of tolerances for $\rho$, $\tau _1$, $\tau _2$, ..., $\tau _T$ (the initialization can be thought of as time step zero, with $\tau _0 = \infty$). The iterative improvement is implemented according to Algorithm 1 below.
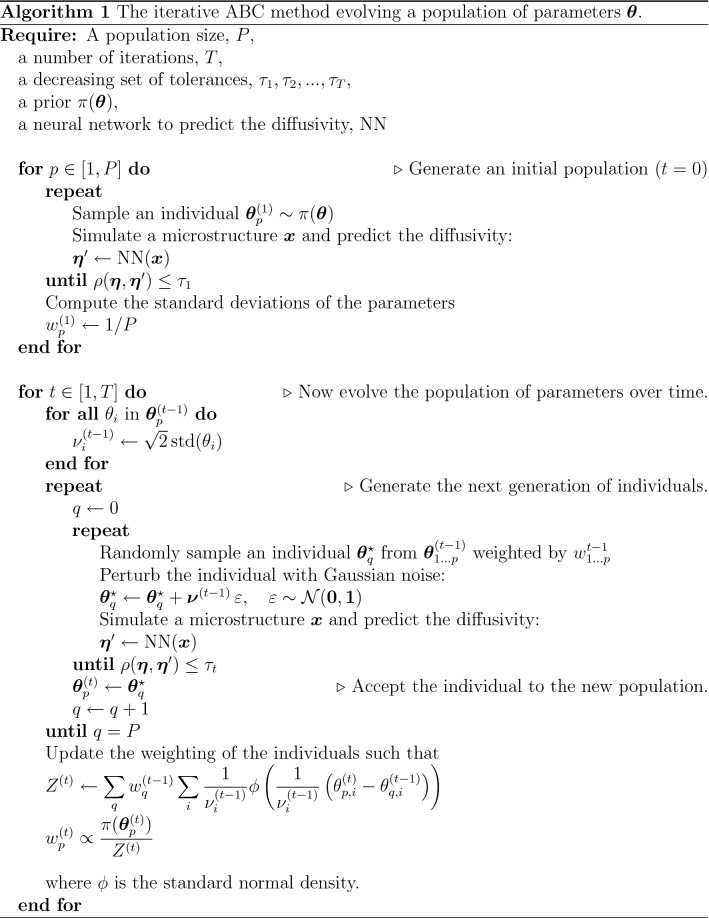


## Data Availability

The datasets generated and/or analysed during the current study are available in the Zenodo repository, 10.5281/zenodo.5778999.
